# First-line therapy in atypical hemolytic uremic syndrome: consideration on infants with a poor prognosis

**DOI:** 10.1186/s13052-014-0101-7

**Published:** 2014-12-11

**Authors:** Nóra Szarvas, Ágnes Szilágyi, Velibor Tasic, Valbona Nushi-Stavileci, Aspazija Sofijanova, Zoran Gucev, Miklós Szabó, Attila Szabó, Lilla Szeifert, György Reusz, Krisztina Rusai, Klaus Arbeiter, Thomas Müller, Zoltán Prohászka

**Affiliations:** 3rd Department of Internal Medicine, Semmelweis University, Kútvölgyi st. 4, H-1125 Budapest, Hungary; Department of Pediatric Nephrology, University Children’s Hospital, Skopje, Macedonia; Department of Nephrology, Pediatric Clinic, University Clinical Centre of Kosova, Prishtina, Republic of Kosovo; 1st Department of Pediatrics, Semmelweis University, Budapest, Hungary; Department of Pediatrics, Medical University Vienna, Vienna, Austria

**Keywords:** Atypical hemolytic uremic syndrome, Infant, Eculizumab, Plasma therapy

## Abstract

**Background:**

Atypical hemolytic uremic syndrome (aHUS) is a rare and heterogeneous disorder. The first line treatment of aHUS is plasma therapy, but in the past few years, the recommendations have changed greatly with the advent of eculizumab, a humanized monoclonal anti C5-antibody. Although recent recommendations suggest using it as a primary treatment for aHUS, important questions have arisen about the necessity of immediate use of eculizumab in all cases. We aimed to draw attention to a specific subgroup of aHUS patients with rapid disease progression and high mortality, in whom plasma therapy may not be feasible.

**Methods:**

We present three pediatric patients of acute complement-mediated HUS with a fatal outcome. Classical and alternative complement pathway activity, levels of complement factors C3, C4, H, B and I, as well as of anti-factor H autoantibody and of ADAMTS13 activity were determined. The coding regions of *CFH, CFI, CD46, THBD, CFB* and *C3* genes were sequenced and the copy number of *CFI, CD46, CFH* and related genes were analyzed.

**Results:**

We found severe activation and consumption of complement components in these patients, furthermore, in one patient we identified a previously not reported mutation in *CFH* (Ser722Stop), supporting the diagnosis of complement-mediated HUS. These patients were not responsive to the FFP therapy, and all cases had fatal outcome.

**Conclusion:**

Taking the heterogeneity and the variable prognosis of atypical HUS into account, we suggest that the immediate use of eculizumab should be considered as first-line therapy in certain small children with complement dysregulation.

## Background

Hemolytic uremic syndrome (HUS) defined by hemolytic anemia, thrombocytopenia, and acute renal failure belongs to the group of thrombotic microangiopathies (TMA) [1]. In the majority of cases Shiga toxin-producing bacteria cause HUS, which is also known as ‘typical’ HUS with a good prognosis and without relapses. However, less often the disease follows a relapsing course with high risk of permanent renal damage or even death [2,3]. In the majority of these cases with this ‘atypical’ HUS (aHUS), severe dysregulation of the alternative complement pathway can be detected, although, there are exceptions to this rule. Mutations, risk haplotypes, and copy-number variations in the genes encoding complement factors (such as complement factor H, membrane cofactor protein, complement factor I, complement C3, complement factor B or thrombomodulin) [1,2,4], and the presence of factor H autoantibodies [5] associated with the homozygous deletion of the complement factor H-related gene 1 [6] have been described as predisposing factors for aHUS [1,2,4]. Much less is known about the pathogenesis of the non-complement-mediated forms of aHUS; however, a recent study, applying a next-generation sequencing-based approach has identified the variations of diacyl-glycerol kinase epsilon as a novel risk factor in such patients [7].

According to the 2009 guideline of the European Pediatric Study Group for HUS [3], the first-line treatment of atypical HUS – i.e. HUS not mediated by enterohaemorrhagic *E. coli* (EHEC), and not associated with *Streptococcus pneumoniae* – should be plasma therapy (plasma exchange or plasma infusions) with early assessment of the clinical response. However, in the past few years new prospects have opened in the management of aHUS with the advent of eculizumab. This humanized monoclonal anti C5-antibody, licensed for the treatment of patients with aHUS in the US in September 2011, and in the EU in November 2011, inhibits C5, thereby preventing the generation of C5a and of the terminal complement complex C5b-9. Recently published clinical trials confirmed the efficacy and safety of eculizumab in plasma-dependent aHUS patients and in posttransplant aHUS recurrence [8,9], and a trial has been applied eculizumab as the first-line treatment of aHUS [10] The availability of eculizumab has increased noticeably during the past few years, and recent recommendations based on the above trials, case reports [11] and recent case series [12] suggest its administration as a primary treatment for patients with aHUS [13]. Recently, however, Ruebner and co-workers have raised an important question about the necessity of the immediate use of eculizumab in all aHUS cases [14]. The authors highlighted the heterogeneity of aHUS and described the illustrative case histories of three children aged less than 18 years with this condition. These patients proved responsive to plasma therapy and made full recovery. The authors recommended a stepwise approach to the management of patients with the aHUS phenotype, and suggested administering eculizumab only to patients likely to have an inherited, complement-mediated disorder and in cases without a good response to plasma therapy after 3–5 days.

While agreeing with this opinion, we would like to draw attention to a specific subgroup of patients with rapidly progressive disease and a poor prognosis, in whom plasma exchange or effective plasma therapy may not be feasible due to young age and small body size. We review the cases of three infants to show that for such patients, prompt treatment with eculizumab would be indicated and urgent complement testing may guide immediate therapeutic decisions.

## Patients and methods

### Patient selection

From January 2008 to June 2013, we investigated 5 patients under the age of 2 years with suspected atypical HUS (non-EHEC-mediated, non-pneumococcal disease). Blood samples obtained during the first acute phase were available from only three infants.

In this study, we present case histories of these selected infants (a 7 month old boy from Kosovo, a newborn from Hungary, and a 2 month old girl from Austria) with complement mediated aHUS and fatal outcome during the first disease flare. Relevant clinical and laboratory data were collected from hospital records. The institutional review board of Semmelweis University approved the design of the study.

### Laboratory analysis

Blood samples were collected during the first acute disease flare. Functional assessment of the activity of the alternative and classical complement pathways was performed along with the measurement of the concentrations of complement factors C3, C4, H, B and I, as well as of anti-factor H IgG autoantibodies, and of ADAMTS13 activity. These laboratory determinations have been described in detail elsewhere [15,16].

To measure the levels of factor H protein (as well as of CFHL-1 protein) variants containing tyrosine (p.Tyr402) or histidine (p.His402) at p.402, serum sample of patient 1 was tested with an allele-specific ELISA method using a HK353 ELISA kit (Hycult Biotech), according to the manufacturer’s instructions.

To screen for mutations, all coding exons of the genes encoding complement factor H (*CFH*), factor I (*CFI*), membrane cofactor protein (*CD46*), thrombomodulin (*THBD*), factor B (*CFB*), C3 (*C3*) and diacyl-glycerol kinase epsilon *(DGKE)* were sequenced following PCR amplification. Primer sequences and PCR conditions are available upon request.

To study the copy number alterations of the genes encoding complement factor H (*CFH*) and related genes (*CFHR1*, *CFHR2*, *CFHR3*, *CFHR5*), complement factor I (*CFI)* and membrane cofactor protein (*CD46)* multiplex ligation-dependent probe amplification (MLPA) was carried out by SALSA MLPA probemixes P236-A3 and P296-A1 (MRC-Holland, Amsterdam, The Netherlands) following the manufacturer’s instructions.

## Results

### Patient 1 (Registry code 193)

The male patient was born in Kosovo at week 39 of an uncomplicated pregnancy, with a birth weight of 3400 g. At the age of 7 months, he was taken to the Children’s Hospital in Prishtina due to sudden onset of pallor, weakness, and anemia. There was neither fever, nor diarrhea. His initial laboratory findings (see Table 1) showed severe anemia and thrombocytopenia with normal renal function and diuresis. Direct antiglobulin test was negative.

**Table 1:** **Laboratory findings at the time when aHUS was confirmed**

	**Patient 1**	**Patient 2**	**Patient 3**
Age	7 months	2 days	2 months
Gender	Male	Male	Female
Hemoglobin (g/L)	69 (120–160)	122 (100–160)	83 (120–150)
Platelet count (G/L)	26 (150–400)	65 (130–450)	28 (140–440)
LDH (U/L)	13500 (<500)	4826 (200–800)	5230 (100–300)
Creatinine (μmol/L)	316 ( <88)	182 (20–105)	98 (8,8-88)
Fragmentocytes	+	+	-
RBC transfusion (Unit)	6 times	2 times	-
Plasma therapy (number of sessions)	FFP (6)	FFP (9)	FFP (1)
Dialysis (sessions)	15	7	2
Outcome	Seizures, coma and respiratory failure, death on day 29 of hospitalization	Pulmonary edema and pneumothorax, death on day 17 of hospitalization	Cerebral edema, brain death on day 3 of hospitalization
Comorbidities	-	Patent ductus arteriosus	Colonic necrosis, coagulopathy

Normal values are between brackets.

He was initially considered to have sepsis and was given antibiotic treatment. He received packed red blood cells and fresh frozen plasma transfusions on three occasions. His hematological parameters improved, but on day 10 of hospitalization, his renal function deteriorated rapidly. He became anuric and decompensated dilated cardiopathy developed, which required hospitalization at the University Children’s Hospital Skopje, for peritoneal dialysis.

Atypical HUS was suspected, but (after plasma therapy) his hematological parameters were inconclusive (haemoglobin level and platelet count were normal) on admission. Fragmented red blood cells could not be demonstrated even by repeated blood smear analysis, and complement C3 level was normal at 0.90 g/L. Peritoneal dialysis improved the patient’s clinical condition and cardiac function. His creatinine level peaked at 316 μmol/L.

On day 19 of hospitalization, his overall condition worsened, severe anemia and seizures occurred. This time, blood smear analysis revealed many schistocytes. Follow-up laboratory tests confirmed the relapse of severe anemia and thrombocytopenia. At this point, the clinical diagnosis of atypical HUS was certain and appropriate blood samples were sent to Hungary for the comprehensive analysis of complement factors. These blood samples, received 24 days after disease onset and after plasma therapy, were tested on the day of arrival of sample for C3, alternative pathway and ADAMTS13 activity, and the results indicated severe complement dysregulation.

The patient was intubated and mechanically ventilated, transfused on three occasions with packed red blood cells and fresh frozen plasma. Despite intensive therapy and the administration of plasma, the baby fell into coma. Respiratory failure accompanied by symptomatic multiorgan failure led to the patient’s death on day 29 of hospitalization. Autopsy was not performed.

### Patient 2 (Registry code 229)

The male neonate was born in Hungary, from an uneventful pregnancy and delivered by the vaginal route, at 36 weeks of gestation, with a birth weight of 2150 g.

On the second day of life, poor feeding, abdominal distension, hepatomegaly, respiratory distress were observed along with reduced skin perfusion and oliguria despite normal blood pressure. Bacterial sepsis was suspected and antibiotic treatment was introduced.

The laboratory tests showed anemia, low platelet count, and metabolic acidosis, as well as bleeding disorder (increased INR; fibrinogen: under detection limit), and a marked elevation of LDH. However, no hyperbilirubinemia was present. On the 4^th^ day of life, uremia, low platelet count, and metabolic acidosis persisted, and moderate increase of alanine aminotransferase and aspartate aminotransferase levels were seen. Direct antiglobulin test was negative.

Progressive circulatory failure required mechanical ventilation and catecholamine treatment from the 4^th^ day of life. Echocardiography depicted a haemodynamically significant, patent ductus arteriosus. The differential diagnosis excluded bacterial sepsis, renal vein thrombosis, and metabolic disorder. The treatment was supplemented with regular, periodic administration of fresh frozen plasma, based on the clinical signs and laboratory results indicative of aHUS.

Progressive circulatory failure, oliguria and uremia culminated in extreme volume overload and pulmonary edema. Therefore, hemofiltration was started on the 9^th^ day of life and repeated altogether 7 times. Despite all these efforts, the patient did not improve and died on the 19^th^ day of life of circulatory failure unresponsive to the treatment.

The serum complement profile obtained on day 14 showed alternative pathway dysregulation and supported the diagnosis of aHUS. Investigations during autopsy could not identify any alternative (infective or malignant) causes of HUS. The histological examination of the kidney and the lung revealed signs of small vessel microangiopathy (thickening of capillary walls, lamellation, detachment of endothelial cells, narrowing of the capillary lumen).

### Patient 3 (Registry code 304)

The female infant was born in Austria, at 38 weeks of gestation with a birth weight of 3750 g. The antenatal and the perinatal periods were uneventful.

At the age of 2 months, she developed acute abdominal distension and cyanosis. There was no history of infection, vomiting, or diarrhea in the previous days.

Initial laboratory findings included high LDH, CK, and serum transaminase levels. Serum albumin concentration was greatly reduced. INR, PTT, and thrombin time were prolonged, C-reactive protein was normal; interleukin-8 level and the leukocyte count were elevated. Renal function parameters, platelet count, and hemoglobin were normal on day 1.

Abdominal ultrasound showed massive edema of the bowels, particularly of the colon and the duodenum. A plain abdominal film did not depict any sign of free air. The progressive decline of oxygen saturation and worsening respiratory insufficiency necessitated respiratory support and then, intubation and mechanical ventilation.

During the next hours, oliguria progressed to anuria, and generalised edema developed. Simultaneously, platelet count and hemoglobin level decreased, whereas renal function parameters increased. Peritoneal dialysis was initiated on day 2 due to progressive edema and renal insufficiency. The follow-up laboratory tests showed further elevation of the LDH level. Complement dysregulation was detected on day 2 (Table 2). The direct antiglobulin test was negative; haptoglobin level was decreased, and free hemoglobin concentration was increased in the plasma. These findings indicated intravasal hemolysis; however, fragmentocytes were absent from the blood smear. Irreversible multiorgan failure developed during the next hours; the CT scan confirmed generalized brain edema, while further testing excluded underlying infectious and/or metabolic causes of the multiorgan failure. The patient died on day 3.

**Table 2:** **Complement and genetic profiles of the members of the three affected families**

	**Patient 1**	**Father 1**	**Mother 1**	**Sister 1**	**Patient 2**	**Father 2**	**Mother 2**	**Patient 3**	**Father 3**	**Mother 3**	**Brother 3**	**Sister 3**
Classical pathway activity CH50/mL (48–103)	**47**	n.d.	n.d.	n.d.	**25**	n.d.	n.d.	**0**	n.d.	n.d.	n.d.	n.d.
Alternative pathway activity % (70–105)	**5**	88	97	108	**0**	71	77	**0**	106	110	70	72
C3 g/L (0.9-1.8)	**0.36**	0.98	1.32	1.23	**0.26**	**0.65**	1.19	**0.41**	1.26	1.42	1.07	1.09
C4 g/L (0.15-0.55)	0.23	n.d.	n.d.	n.d.	**0.1**	n.d.	n.d.	**0.05**	n.d.	n.d.	n.d.	n.d.
Factor H:Ag mg/L (250-880)	**<95**	378	813	631	**123**	369	549	**<95**	374	685	431	338
Factor B % (70–130)	**65**	81	98	90	**50**	**52**	100	**19**	**60**	98	70	90
Factor I % (70–130)	**42**	108	108	104	**47**	**69**	112	**26**	85	112	72	81
Anti-factor H IgG autoantibody	negative	n.d.	n.d.	n.d.	negative	n.d.	n.d.	negative	n.d.	n.d.	n.d.	n.d.
ADAMTS13 activity (67-151%)	**27**	214.	143	171	**9**	133	101	**8**	124	112	142	157
ADAMTS13 inhibitor	negative	n.d	n.d	n.d	n.d	n.d	n.d	negative	n.d	n.d	n.d	n.d
Mutation (Numbering from Met1)	het CFH Ser722Stop	het CFH Ser722Stop	-	-	-	-	-	-	-	-	-	-
aHUS risk haplotypes	het CFH H3; het MCPggaac	het MCPggaac	het CFH H3	het CFH H3	-	-	het CFH H3; het MCPggaac	het MCPggaac	hom MCPggaac	het MCPggaac	het MCPggaac	het MCPggaac
Missense variations	het CFH Y402H; het CFH E936D; het C3 R102G; het C3 P314L; het CFB R32W	hom CFH Y402H; het C3 R102G; het C3 P314L	het CFH Y402H; het CFH E936D; het CFB R32W	het CFH Y402H; het CFH E936D;	hom CFH Y402H; het CFB R32W	hom CFH Y402H; het CFB R32W	het CFH Y402H; het CFH E936D;	hom CFH Y402H; het C3 R102G; het C3 P314L; het CFB L9H	het CFH V62I, het CFH Y402H; hom C3 R102G; hom C3 P314L; het CFB L9H	hom CFH Y402H; het CFB R32Q	het CFH V62I, het CFH Y402H; het C3 R102G; het C3 P314L; het CFB R32Q	hom CFH Y402H; het C3 R102G; het C3 P314L

*Abbreviations* used: *het* heterozygous, *hom* homozygous, *n.d.* not determined, bold characters indicate values below the reference range.

Investigations during autopsy could not identify any alternative (infective or malignant) causes of HUS. The histological examination of the kidney, spleen, lung, bowel, and myocardium confirmed thrombotic microangiopathy.

### The results of complement- and genetic testing

The detailed results of complement profiling are presented in Table 2. Alternative complement pathway activity was decreased in all three patients. The consumption of C3, as well as decreased levels of complement factor B and I were found in all cases. Factor H level was below the detection limit in patient 1 and 3, and it was decreased in patient 2. The level of C4 and the activity of the classical pathway were decreased in patients 2 and 3. Since we observed unusual concomitant decrease in these factors in patients 2 and 3, we have measured the levels of potassium, magnesium, calcium and phosphate in these serum samples and none of them was decreased excluding the possibility that samples were diluted with infusion.

ADAMTS13 activity was decreased but not deficient in all cases, and the presence of the ADAMTS13 inhibitor was excluded in two cases (Table 2). None of the family members had low (<67%) ADAMTS13 level indicative of ADAMTS13 deficiency. We suspect secondary ADAMTS13 consumption by the ultra-large von Willebrand factor due to extensive endothelial activation.

All patients were negative for anti-factor H autoantibody. Regarding the family members of patients, no signs of complement deficiency or dysregulation was detected in the family of patient 1. The father of patient 2 had low levels of factor B, I and C3, the father of patient 3 had below normal level of factor B, while other family members in these families had normal complement parameters.

To investigate the genetic alterations in our patients, we sequenced the coding regions of complement genes *CFH, CFI, CD46, THBD, CFB*, *C3 and DGKE*. Patient 1 was heterozygous for a cytosine to adenine substitution (c.2165C>A) in exon 15 of *CFH* (encoding short consensus repeat 12) that causes a serine to STOP change at p.722 (Ser722Stop) in the protein. Mutation at this site has not been reported previously. Based on the analysis of inheritance in the patient’s family, this mutation was located on an allele coding histidine at p.402. As the patient was heterozygous for the Tyr402His (Y402H) polymorphism, we applied an ELISA test system using two different monoclonal antibodies, specific for the His402- and Tyr402- containing factor H proteins (HK353 ELISA kit, Hycult Biotech). The level of factor H protein from the Ser722Stop mutation-containing allele (p.402His-containing factor H) was below the lower limit of quantification, while the level of factor H from the other allele (p.402Tyr-containing factor H) was low, but still measurable in the serum sample of the patient. The mutation-carrying father of the patient was not heterozygous for the Tyr402His polymorphism, therefore there are no comparable alleles in his serum, so we did not apply this method in this case. Based on these results we concluded that the mutation p.Ser722Stop by causing the premature termination of translation presumably leads to deficient synthesis and/or secretion of the mutated protein. No disease-causing mutation was detected in the other two patients.

Sequencing of the selected complement genes revealed the presence of multiple polymorphic variants. As inferred from genotype data, one patient (patient 1) carried the H3 risk haplotype of the *CFH* gene, and two patients (patient 1 and patient 3) carried risk alleles of MCP polymorphisms (MCPggaac). The results of the pedigree analysis are presented in Table 2.

To reveal deletions or duplications that may influence disease development, *CFI, CD46* (MCP) as well as *CFH* and its related genes were studied by applying MLPA probemixes of MRC-Holland. None of the patients showed copy number alterations in the selected genes.

## Discussion

Recent findings on the pathogenesis and the new therapeutic options have markedly changed the recommendations on the initial investigations and therapy of choice in aHUS. However, a number of questions remain open regarding the optimal management of patients with aHUS [17]. We presented this small series of three patients with acute complement-mediated HUS to highlight a specific subgroup of infants characterized by rapid disease progression and high mortality, as well as to pinpoint the important aspects of the clinical management of these cases.

The classification of HUS as atypical HUS is based on the clinical signs (see the 2009 guideline), and the recognition of HUS in the context of sepsis or disseminated intravascular coagulation is often delayed, because anemia and thrombocytopenia are characteristic of both diseases. Plasma therapy may interfere with the laboratory tests used to support aHUS and can thereby delay correct diagnosis. However, initial complement results may be helpful and these can be obtained within 1–2 days. According to the Clinical Practice Guidelines for the management of aHUS [3], detailed complement investigation is recommended in all cases at the onset of the episodes, before administering plasma therapy. To explore the complement-related pathogenetic factors, it is suggested to test the serum levels of C3, C4 (for differential diagnosis), factor I and factor H, as well as of autoantibodies to factor H and MCP expression on leukocytes. The genetic analysis of selected complement factors should also be performed. During the recent years, we added two more tests to the diagnostic evaluation of the alternative complement pathway, i.e. the measurement of total AP activity, and of factor B level. According to our previous results [18], multiple assessment of the components and total activity of the alternative pathway (decreased C3, factor B and alternative pathway activity) appears to yield specific information on complement-mediated processes in aHUS.

**Figure 1:**
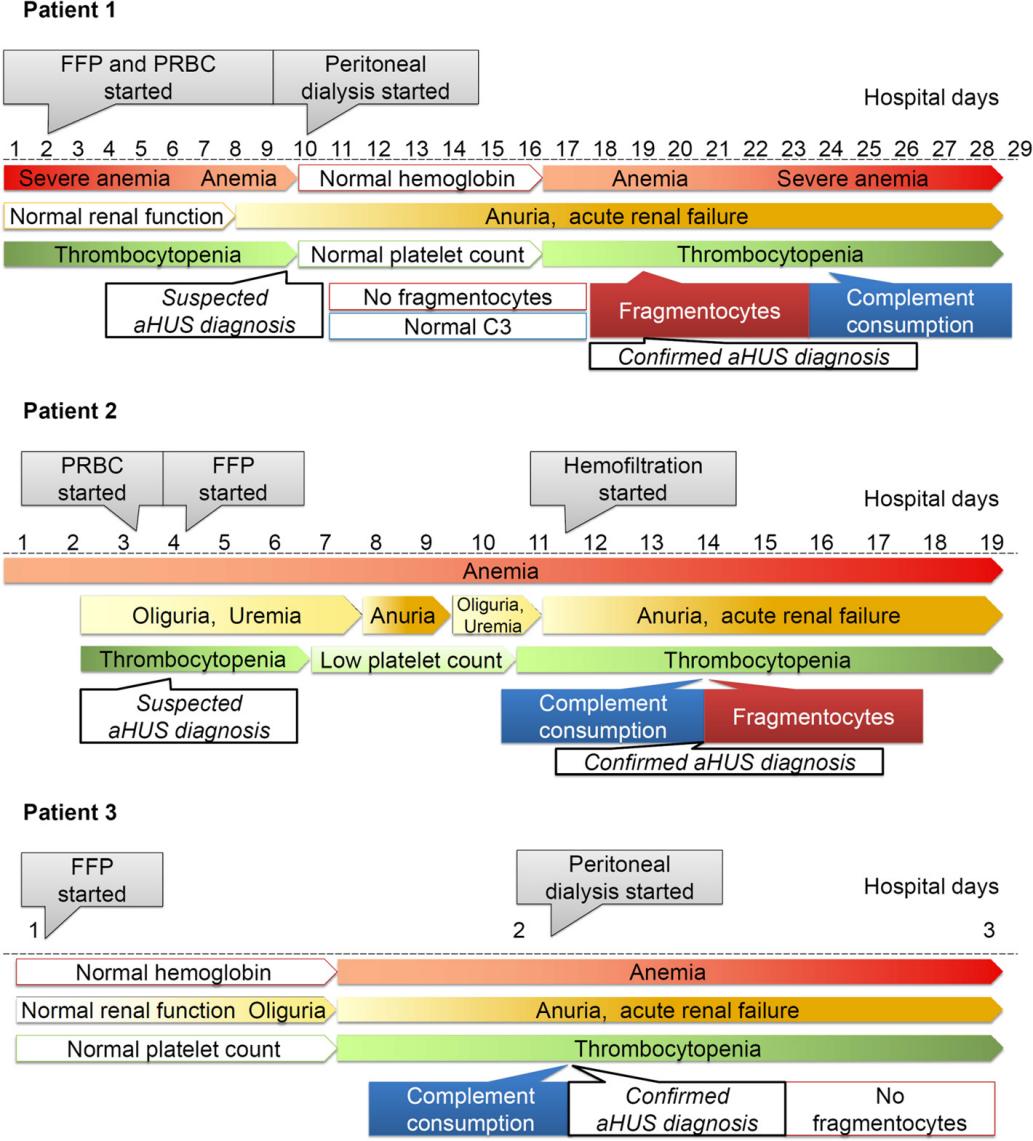
**Development of symptoms and laboratory signs of aHUS during the course of disease in the three patients.** Abbreviation used: PRBC: packed red blood cells; FFP: Fresh frozen plasma; C3: Complement C3. (Microsoft Office PowerPoint 97–2003 was used to create the artwork).

In this small series of cases with clinically diagnosed aHUS, all patients had decreased alternative pathway activity with consumption of factors C3 and B, as well as with unusually low levels of factors H and I, indicating extensive amplification and consumption of the alternative pathway with its regulators. In our opinion, all three patients had complement-mediated HUS, as all of them presented with hemolytic anemia, thrombocytopenia and acute renal failure (Figure 1). Moreover, severe dysregulation and consumption of the alternative complement pathway could be verified within a day after the arrival of the blood sample to the laboratory. It should be noted, however, that the marked decrease of classical pathway activity, C4 levels, and ADAMTS13 activity in our cases indicates an uncommon pathogenetic mechanism (most likely induced by yet unidentified genetic predisposing factors in cases 2 and 3). Low but not deficient ADAMTS13 level of patients besides the absence of ADAMTS13 inhibitors (where measured) excluded the diagnosis of TTP, while based on the normal levels of ADAMTS13 activity of the healthy family members we ruled out the possibility of Upshaw-Schulman syndrome. Furthermore, unidentified, alternative causes of HUS, such as infectious, malignant, immunologic, or metabolic diseases may also be suspected in our patients. However, the fact that no such diseases were found during autopsy in patients 2 and 3, renders this possibility improbable. The findings of the histological investigations performed during autopsy supported the diagnosis of TMA in patients 2 and 3. Therefore we consider that complement mediated HUS was the primary diagnosis in these cases. Since signs of coagulation disorder were also present disseminated intravascular coagulation accompanied aHUS in patients 2 and 3.

Analyzing the predisposing genetic components within complement factors, we identified combined risk factors (factor H mutation Ser722Stop and two risk haplotypes in *CFH* and *MCP*) for patient 1, and non-synonymous SNPs (in *C3* or *CFB*) and/or aHUS risk haplotype (MCPggaac) for patients 2 and 3. In approximately 40-50% of aHUS cases, no mutations can be identified in the complement genes [2,19]. The missense polymorphisms of C3 (rs2230199: R102G, rs1047286: P314L) and factor B (rs4151667: L9H and rs12614: R32W) identified in the patients has unknown significance in aHUS. However, carriers of the rare alleles of the R102G SNP and of the P314L polymorphism as well as of their haplotype were reported to have lower AP50 values (higher alternative pathway activity) than those with homozygous wild genotypes [20]. In agreement with this, in hemolysis assays G102 activated the alternative pathway more efficiently and had higher hemolytic activity compared with the R102 C3 protein as in the presence of glycine at p.102 the efficiency of factor H as a cofactor in C3b inactivation was reduced [19]. Since decreased levels of complement parameters (predominantly factor B) were observed in healthy family members (i.e. in the father of patients 2 and 3), we suspect additional predisposing genetic variants in the background of the disease [4].

Since the publication of the “European Guideline for the investigation and initial therapy of HUS” significant progress has been made in the therapy of HUS with the clinical verification of the efficacy of eculizumab [8], an anti-C5 monoclonal antibody based inhibitor of the complement terminal pathway. Physicians treating patients with atypical HUS have to choose between plasma therapy and eculizumab for the initial treatment of acute disease.

Although the guidelines recommend plasma exchange [1,3], low total blood volume and difficult vascular access make plasmapheresis technically challenging in infants; therefore, this therapeutic option is seldom used [21,22]. Fresh frozen plasma transfusion is an alternative option, but this treatment will not eliminate potentially harmful components and as in our cases, patients often do not respond to FFP. Moreover, mortality rate appears to be the highest in patients under 2 years [19,23]. Based on a recent paper, there were 9 cases with fatal outcome and 6 of them were under 2 years of age in the French cohort. In the Dutch cohort, only the youngest patient in the study, a one month old baby died during the first aHUS episode. In accordance with these data, we have also observed a high mortality rate in this age group: altogether 4 of our 5 infant patients died under the age of 1 (unpublished data).

We report these patients to draw attention to a plasma-resistant disease subtype with a severe outcome in infants. We believe that eculizumab might have saved the lives of our patients, but this therapy could not be started fast enough in our cases due to its high price (in Hungary), its unavailability (in Macedonia), or because of the rapid progression of disease (in Austria). Previously, other reports confirmed that eculizumab could be an effective therapy for infants with aHUS of rapidly progressive onset, when plasmapheresis is not feasible [10,24-26]. In agreement with these recommendations [10,17,26], we also suggest that eculizumab should be the first-line therapy for infants with hypocomplementemia accompanied by the clinical symptoms of aHUS. Treatment should start immediately when clinical diagnosis of HUS can be verified and the atypical form is suspected [3]. A recently published paper proposed a stepwise approach for the management of aHUS [14]. This would be appropriate for most patients; however, in the case of infants, we would not be willing to wait 3–5 days to assess the patient’s response to plasma therapy. Urgent investigation of the alternative complement pathway is appropriate to stratify patients for eculizumab therapy. Therefore, in agreement with a recent recommendation [17], we would suggest prompt treatment with eculizumab in small pediatric cases, once complement dysregulation has been recognized.

## Conclusion

Infants constitute a specific subgroup of atypical HUS patients, with rapid disease progression and high mortality, in whom plasma therapy may not be feasible. The use of eculizumab should be considered as first-line therapy in these pediatric cases. Urgent investigation of the alternative complement pathway is suitable to stratify patients for eculizumab therapy. We would suggest that the treatment should start immediately, once the diagnosis of HUS has been verified and the atypical form is suspected.

## Consent

The parents of patients gave their consent to the publication of the report.
